# Glycyrrhizic Acid Attenuates Sepsis-Induced Acute Kidney Injury by Inhibiting NF-*κ*B Signaling Pathway

**DOI:** 10.1155/2016/8219287

**Published:** 2016-01-24

**Authors:** Hongyu Zhao, Min Zhao, Yu Wang, Fengchun Li, Zhigang Zhang

**Affiliations:** Department of Emergency Medicine, Shengjing Hospital of China Medical University, 36 Sanhao Street, Shenyang 110004, China

## Abstract

Glycyrrhizic acid (GA) is a major active ingredient in licorice. In our study, the effects of GA on acute kidney injury (AKI) in rats and its underlying molecular mechanisms were investigated. The sepsis model was produced by caecal ligation and puncture (CLP) in rats. The molecular and histological experiments were performed in the kidney tissues and serum samples of rats. According to the results obtained, GA alleviated sepsis-induced AKI by improving the pathological changes, decreasing the levels of blood urea nitrogen (BUN), creatinine (Cre), and increasing the survival rate of rats with AKI significantly. The production of inflammatory cytokines, such as TNF-*α*, IL-1*β*, and IL-6, was markedly inhibited by GA. Moreover, treatment with GA inhibited the production of nitric oxide (NO) and prostaglandin E2 (PGE2) and expression levels of induced nitric oxide synthase (iNOS) and cyclooxygenase-2 (COX-2) in kidney tissues. Furtherly, the apoptosis in kidney tissue induced by AKI was suppressed by GA. Finally, GA could inhibit the activation of NF-*κ*B signaling pathway. Our study suggests that GA alleviates sepsis-induced AKI by inhibiting the NF-*κ*B signaling pathway, which provides a strong evidence for a new approach for treating sepsis-induced AKI.

## 1. Introduction

Sepsis is a systemic inflammatory response syndrome (SIRS) induced by infection [1]. Despite improvement in the clinical technology for intensive care, the mortality rate induced by sepsis in intensive care units remains high [2–4]. The latest epidemiological studies show that each year more than 18 million people are infected by severe sepsis in the world and the number is rising at an annual rate of 1.5%~8.0%. The mortality rate is especially remarkable, ranging up to 60%. Sepsis, in severe cases, may progress to organ failure and death [5]. About 42% patients with sepsis may experience AKI [6], and AKI is an independent factor of mortality in sepsis. Since the mortality rate is as high as 70% [7], sepsis-induced AKI is one of the critical urgent problems in critical care medicine and nephrology. So far, it has been known that there are many aspects about the pathophysiological mechanisms of sepsis-induced AKI: vasodilation-induced glomerular hypoperfusion, dysregulated circulation within the peritubular capillary network, apoptosis of kidney cells, inflammatory reactions by cytokines overproduction, and oxidative stress-induced tubular dysfunction [8].

GA, a primary bioactive constituent of the shrub* Glycyrrhiza glabra*'s roots, has strong and extensive effects on immunomodulatory, antiparasitic, antioxidant, antiviral, and antitumor activities [9–13]. At present, GA is widely used in Asia as a therapeutic agent for chronic viral hepatitis [14, 15]. Recent studies also have shown that GA has a variety of protective effects on kidney. For example, research found that GA had therapeutic effect on experimental obstructive nephropathy in rats [16]. In addition, GA also can protect renal tubular epithelial cells against injury induced by high glucose [17]. The study by Bi et al. has also shown that glycyrrhizin had protective effect on nephrotic syndrome induced by adriamycin in rats [18]. However, the effect of GA on sepsis-induced AKI and its related molecular mechanisms have been poorly understood so far.

In the present study, we aim at observing the beneficial effect of GA on sepsis-induced AKI and analyzing the detailed molecular mechanisms in a sepsis rat model by caecal ligation and puncture (CLP), which is recognized as having strong clinical relevance [19].

## 2. Materials and Methods

### 2.1. Drugs and Antibodies

**Figure 1:**
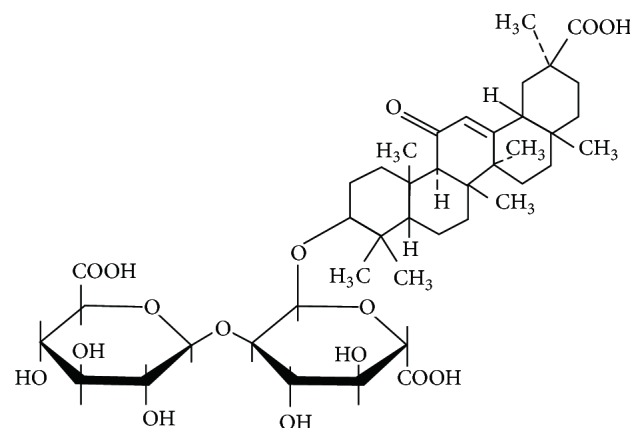
Chemical structure of GA. The molecular formula of GA is C_42_H_62_O_16_ and its molecular weight is 822.93.

GA was purchased from Meilun Biological Co., Ltd. (Dalian, China), and dissolved in 10% DMSO at a concentration of 5 mg/mL. The chemical structure of GA is shown in Figure 1. The antibodies used for western blotting and immunohistochemistry were as follows: anti-Bcl-2 (Boster, China), anti-Bax (Boster, China), anti-I*κ*B*α* (Bioss, China), anti-cleaved caspase-3 (Abcam, USA), anti-NF-*κ*B (Boster, China), anti-p-NF-*κ*B (Bioss, China), anti-Cox-2 (Boster, China), anti-iNOS (Bioss, China), anti-*β*-actin (Boster, China), and anti-Histone H3 (Bioss, China).

### 2.2. Animals and Experimental Protocol

Pathogen-free male Sprague-Dawley rats (weight 200 ± 20 g) were purchased from Beijing Vital River Laboratory Animal Co., Ltd. (Beijing, China). The animals were maintained at 23°C under a 12 h light-dark cycle with free access to food and water. The night before the experiments, animals had no access to food but gained free access to water. 50 rats were randomly divided into five experimental groups (*n* = 10 per group): sham operation group, GA (50 mg/kg) group, sepsis group, sepsis plus GA (25 mg/kg), and sepsis plus GA (50 mg/kg). We constructed sepsis model by CLP. Briefly, the rats were anaesthetized by injection of sodium pentobarbital (50 mg/kg) and a laparotomy was performed through a 2 cm ventral midline abdominal incision. We punctured the cecum twice at different sites with an 18-gauge needle and gently compressed until faces were extruded and then repositioned it. The incision was closed in 2 layers. The sham operation group underwent laparotomy through a midline incision, but the cecum was not punctured. Animals in the GA (50 mg/kg) group, sepsis plus GA (25 mg/kg) group, and sepsis plus GA (50 mg/kg) group were intraperitoneally injected with GA 25 mg/kg or 50 mg/kg, while sham operation group and sepsis group were intraperitoneally injected with isovolumetric normal saline. 24 h after surgery, all the animals were euthanized and peripheral blood and kidney tissues were collected for further tests. All animal experiments were carried out strictly in accordance with international ethical guidelines and the National Institutes of Health Guide concerning the Care and Use of Laboratory Animals. The experiments were approved by the Institutional Animal Care and Use Committee of Shengjing Hospital of China Medical University.

### 2.3. Survival Curves

To observe the effect of GA on survival, another 40 rats were randomly divided into four experimental groups (*n* = 10 per group): sham operation group, sepsis group, sepsis plus GA (25 mg/kg) group, and sepsis plus GA (50 mg/kg) group. The observation of survival was performed every 12 h until the endpoint at 96 h.

### 2.4. Periodic Acid-Schiff (PAS) Staining

The kidney tissue samples were fixed in 10% buffered formalin for 48 h and were then dehydrated by washing with ascending grades of ethanol. Then, samples were sectioned and embedded in paraffin wax. 5 *μ*m kidney sections were prepared for routine PAS stains. The morphologic change of kidney was assessed by staining with PAS. Under 400x magnification, each section was randomly selected and photographed for 5 fields. The kidney injury was assessed by the following criteria, referred to in previous method [20]: 0, normal; 1, damage involving less than 25% of the area; 2, damage involving 25% to 50% of the area; 3, damage involving 50% to 75% of the area; 4, 75% to 100% of the area being affected.

### 2.5. Serum Analysis

Blood samples collected following CLP were used for the detection of blood BUN and Cre levels using commercially available kits produced by Jiancheng Bioengineering Institute (Nanjing, China), following the instructions of the manufacturer. The concentrations of BUN and Cre were calculated by generating a standard curve.

### 2.6. Inflammatory Cytokine Measurement in Kidney

The levels of TNF-*α*, IL-1*β*, and IL-6 in rats kidney tissues were measured by commercially available ELISA kits (Wanleibio, China) according to the manufacturer's instructions. The concentrations of TNF-*α*, IL-1*β*, and IL-6 were determined by a standard curve and expressed as pg/mL.

### 2.7. Determination of NO

The amount of nitrite (a stable metabolite of NO) in the kidney tissues was detected by the Griess Reagent System (Beyotime, China) according to the manufacturer's recommendation. Absorbance was measured at 540 nm and the nitrite concentration was determined using various kalium nitrite concentrations (1, 2, 5, 10, 20, and 50 *μ*M) as a standard.

### 2.8. Determination of PGE_2_

The level of PGE_2_ in the kidney tissues was detected by commercially available ELISA kit (Wanlei, China) according to the manufacturer's recommendation. The concentration of PGE_2_ was determined by a standard curve and expressed as pg/mL.

### 2.9. Terminal Deoxynucleotidyl Transferase-Mediated dUTP Nick End Labelling (TUNEL) Staining

The presence of apoptotic cells in kidney tissues was detected by TUNEL on rat kidney tissue sections using the DeadEnd Fluorometric TUNEL System (Promega, USA) following the manufacturer's protocols. The numbers of TUNEL-positive cells were counted from six sections under a microscope (400x).

### 2.10. Western Blot Analysis

Total proteins and nuclear proteins in the kidney tissues were extracted by commercially available kits (Beyotime, China) and then denatured. The protein concentration was determined by the BCA protein estimation kit (Beyotime, China). Equal quantities proteins samples were separated by SDS-PAGE and then transferred to polyvinylidene fluoride. The membranes were blocked with 5% nonfat dry milk in PBS and incubated with anti-Bcl-2 (1 : 1000), anti-Bax (1 : 1000), anti-cleaved caspase-3 (1 : 1000), anti-I*κ*B*α* (1 : 1000), anti-NF-*κ*B (1 : 1000), anti-p-NF-*κ*B (1 : 1000), anti-Cox-2 (1 : 1000), anti-iNOS (1 : 1000), anti-*β*-actin (1 : 1000), and anti-Histone H3 (1 : 1000) antibodies, respectively, at 4°C overnight. The goat anti-rabbit secondary antibody conjugated with horseradish peroxidase (1 : 5000) was incubated. Results were carried out with ECL detection reagent (Beyotime, China). Photographs were taken and the optical densities of the bands were scanned and quantified with the Gel Doc 2000 (BioRad, USA).

### 2.11. Immunohistochemistry

The expressions of iNOS and COX-2 from different groups kidney tissues were detected by immunohistochemistry. The kidney tissues were fixed in 4% paraformaldehyde and then imbedded in paraffin. Kidney sections of 5 *μ*m were sectioned and placed on poly-L-lysine-coated slides and then kept in an oven at 60°C for 24 h to increase section adherence to the slide. The slides were deparaffinized using xylene and dehydrated by graded concentrations of alcohol, then placed in a retrieval solution, and incubated in a microwave at high power for 15 min. The slides were then cooled and washed with wash buffer and incubated sequentially with primary antibody and biotin-labeled secondary antibody. Finally, the sections were stained with DAB, counterstained with hematoxylin, dehydrated, cleared in xylene, and fixed. Under 400x magnification, 5 different microscopic fields were randomly chosen.

### 2.12. Electrophoretic Mobility Shift Assay (EMSA)

The tissues were homogenized and nuclear protein was extracted with a nuclear and cytoplasmic kit (Beyotime, China). Protein concentration was determined by the BCA protein estimation kit (Beyotime, China). The level of NF-*κ*B in nucleus was then measured by EMSA. Briefly, nuclear protein samples were mixed with labeled DNA probe. The samples were loaded and run at 180 V for 80 min. Following electrophoresis, protein was transferred from the gel to a positively charged nylon membrane by electroblotting. UV-cross-linked and biotin-labelled DNA was detected by chemiluminescence.

### 2.13. Statistical Analysis

All data are expressed as means ± SD. One-way ANOVA followed by Bonferroni's post hoc test was used for comparisons among experiment groups. Survival data were analyzed utilizing log-rank or *χ* ^2^. A *P* value of less than 0.05 was considered statistically significant.

## 3. Results

### 3.1. Effect of GA against Sepsis-Induced AKI

**Figure 2:**
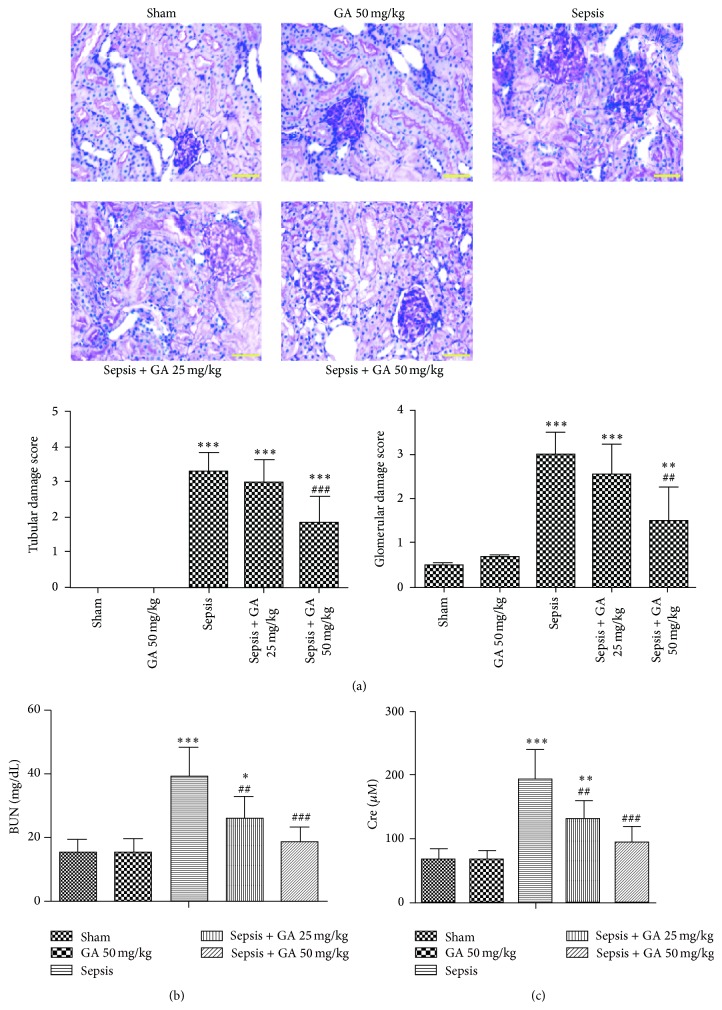
Protective effect of GA against sepsis-induced AKI and liver damage. (a) The pathological changes of kidney tissue were detected by PAS staining assay (magnification 400x) and the tubular injury score was shown. The serum concentrations of BUN (b) and Cre (c) from different groups. The results shown are representative of at least three independent experiments. Each value represents the mean ± SD (*n* = 6). ^*∗*^ *P* < 0.05; ^*∗∗*^ *P* < 0.01; ^*∗∗∗*^ *P* < 0.001, versus the sham operation group.  ^##^ *P* < 0.01; ^###^ *P* < 0.001, versus the sepsis group.

To evaluate the histopathological morphologic changes of kidney, PAS staining assay was performed. As shown in Figure 2(a), the array of the epithelial cells of the proximal tubules is disorderly and obvious drop of epithelial cells could be seen, which resulted in the high tubular damage score in sepsis group. The glomerular volume became bigger, and mesangial cells showed swelling and glassy degeneration. However, GA restrained the pathological changes effectively. Moreover, serum BUN and Cre levels were measured to assess the overall kidney function. The results showed that the levels of BUN and Cre were significantly increased in sepsis group; however, GA could reduce them markedly (Figures 2(b) and 2(c)).

### 3.2. Effect of GA on the Production of Inflammatory Cytokines

**Figure 3:**
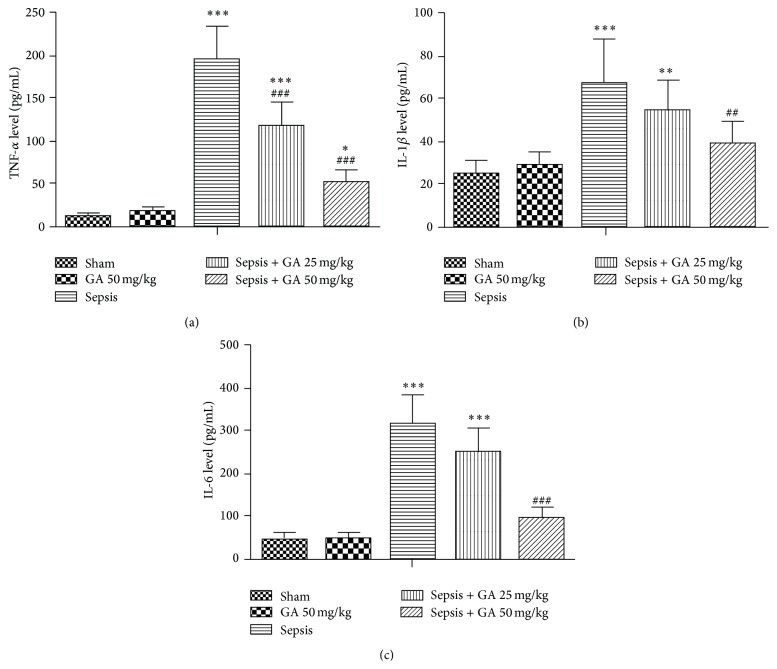
Effect of GA on inflammatory cytokines production. The TNF-*α* (a), IL-1*β* (b), and IL-6 (c) levels in kidney were determined by ELISA. The results shown are representative of at least three independent experiments. Each value represents the mean ± SD (*n* = 6). ^*∗*^ *P* < 0.05; ^*∗∗*^ *P* < 0.01; ^*∗∗∗*^ *P* < 0.001, versus the sham operation group.  ^##^ *P* < 0.01; ^###^ *P* < 0.001, versus the sepsis group.

Since the essence of sepsis is the inflammatory reactions, we detected the productions of inflammatory cytokines, such as TNF-*α*, IL-1*β*, and IL-6, in kidney tissue by ELISA. As shown in Figure 3, the productions of TNF-*α*, IL-1*β*, and IL-6 were increased, induced by AKI, while treatment with GA could inhibit the excess productions of TNF-*α*, IL-1*β*, and IL-6.

### 3.3. Effect of GA on the Productions of NO and PGE_2_ and the Expressions of iNOS and COX-2

**Figure 4:**
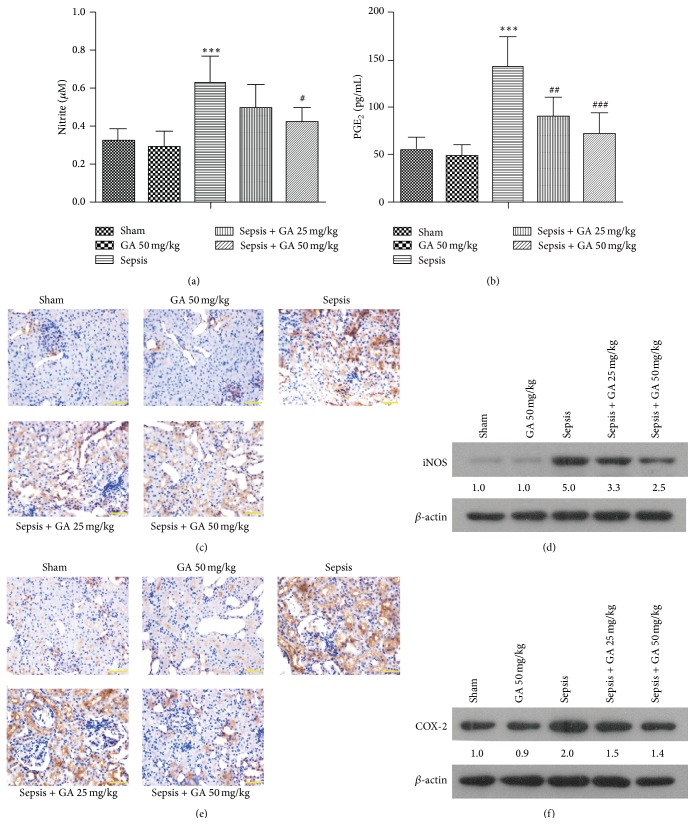
GA inhibited the productions of NO and PGE_2_ and expressions of iNOS and COX-2 in kidney tissue induced by AKI. (a) The amount of nitrite in the kidney tissue was detected by the Griess Reagent System. (b) The concentration of PGE_2_ in kidney from different groups. The expression of iNOS in kidney was measured by immunohistochemical staining (magnification 400x) (b) and western blot (c). The expression of COX-2 in kidney was measured by immunohistochemical staining (magnification 400x) (d) and western blot (e). The results shown are representative of at least three independent experiments. Each value represents the mean ± SD (*n* = 6). ^*∗∗∗*^ *P* < 0.001, versus the sham operation group. ^#^ *P* < 0.05; ^##^ *P* < 0.01; ^###^ *P* < 0.001, versus the sepsis group.

To further justify the effect of GA on inflammatory reactions, the inflammatory mediators productions and proteins expressions were detected. As shown in Figure 4(a), sepsis resulted in a significant increase in NO production in kidney tissue compared with sham group, whereas GA significantly inhibited sepsis-induced production of NO. Moreover, immunohistochemical staining and western blot assay were used to evaluate the expression of iNOS. As shown in Figures 4(c) and 4(d), GA markedly inhibited sepsis-induced expression of iNOS in the same manner as it inhibited the production of NO.

Since COX-2 is one of the downstream targets of NO, which promotes the production of PGE_2_, the production of PGE_2_ and expression of COX-2 were evaluated subsequently. Our result showed that a significant increase in the production of PGE_2_ compared with sham operation group was induced by sepsis, whereas treatment with GA markedly inhibited sepsis-induced production of PGE_2_ (Figure 4(b)). Similarly, GA markedly inhibited sepsis-induced expression of COX-2 by immunohistochemical staining and western blot (Figures 4(e) and 4(f)).

### 3.4. Effect of GA on Renal Cell Apoptosis Induced by Sepsis

**Figure 5:**
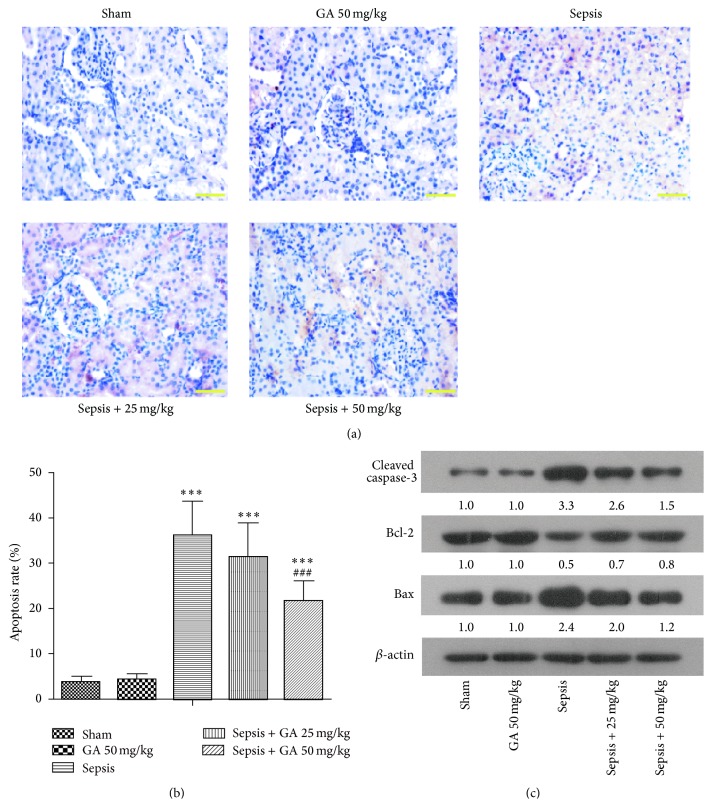
GA inhibited the apoptosis induced by AKI in kidney tissue. (a) The apoptosis of cells in kidney tissue was detected by TUNEL staining (magnification 400x). (b) The apoptosis rate of TUNEL staining from different groups was calculated. (c) Apoptosis-related proteins were detected by western blot. *β*-actin was used as a loading control. The results shown are representative of at least three independent experiments. Each value represents the mean ± SD (*n* = 6). ^*∗∗∗*^ *P* < 0.001, versus the sham operation group. ^###^ *P* < 0.001 versus the sepsis group.

The apoptosis of kidney tissue was detected by TUNEL staining. As shown in Figure 5(a), compared with the sham group, the number of TUNEL-positive cells was increased significantly in the sepsis group, which could be inhibited by GA treatment. The statistics for each group were shown in Figure 5(b). To further confirm the effect of GA against renal cell apoptosis, a number of apoptosis-related proteins in rat kidney were determined by western blot. As shown in Figure 5(c), in sepsis group, the expression of cleaved caspase-3 protein was upregulated. The downregulation of Bcl-2 and upregulation of Bax resulted in a significant decrease in the ratio of Bcl-2/Bax induced by sepsis. However, treatment with GA could downregulate the expression of cleaved caspase-3 and upregulate the ratio of Bcl-2/Bax, compared with sepsis group.

### 3.5. GA Negatively Regulated the Activation of NF-*κ*B Induced by AKI

**Figure 6:**
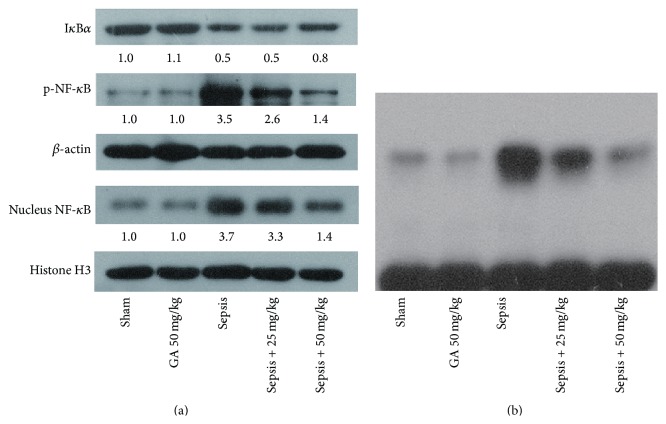
GA inhibited the activation of NF-*κ*B induced by AKI. (a) The expression of NF-*κ*B signaling pathway related proteins was detected by western blot. *β*-actin and Histone H3 were used as a loading control. (b) The transcription activity of NF-*κ*B inhibited by GA in kidney tissue was detected by EMSA assay. The results shown are representative of at least three independent experiments.

NF-*κ*B, a key transcriptional regulatory factor, participates in inflammatory reactions and plays important roles in the disease course. So various assays were adopted to evaluate the effects of GA on the activation of NF-*κ*B. As assessed by western blot and shown in Figure 6(a), we found that the expression of I*κ*B*α* was decreased, while the expressions of p-NF-*κ*B and NF-*κ*B in nucleus were increased significantly in sepsis group, compared with sham group, which demonstrated the activation of NF-*κ*B induced by AKI. However, these changes induced by AKI were inverted by GA treatment. Moreover, EMSA assay was performed to further demonstrate the role of GA in regulating the activity of NF-*κ*B binding to DNA. The EMSA results clearly showed the activation of NF-*κ*B in the sepsis group, while GA could significantly inhibit the activation of NF-*κ*B (Figure 6(b)).

### 3.6. Effect of GA on Survival Rate

**Figure 7:**
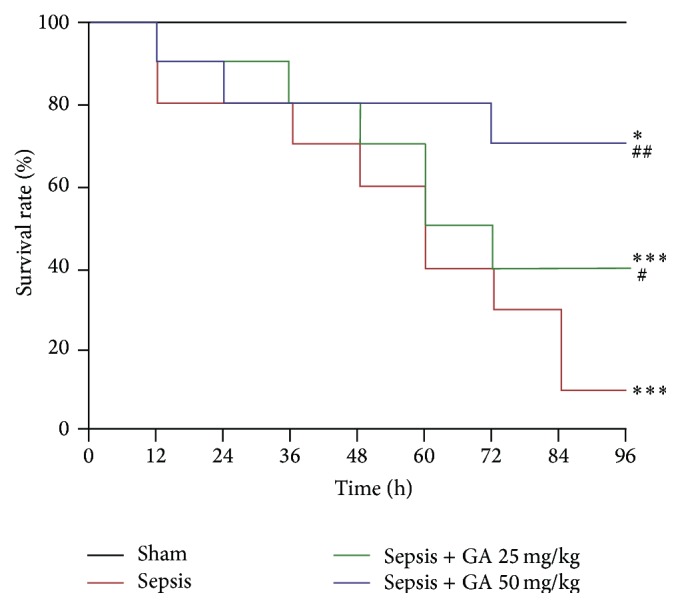
The survival rate of rats in different treatment groups. The survival curves of rats (*n* = 10) with indicated treatment were monitored. ^*∗*^ *P* < 0.05; ^*∗∗∗*^ *P* < 0.001, versus the sham operation group. ^#^ *P* < 0.05; ^##^ *P* < 0.01, versus the sepsis group.

To further confirm the protective effect of GA on sepsis-induced AKI, we detected the survival rate of rats in different treatment groups. As shown in Figure 7, the survival rate of rats in sepsis-induced AKI was significantly decreased compared with sham operation group, which was greatly improved by GA treatment, confirming the effectiveness of GA furtherly.

## 4. Discussion

AKI is a common complication of sepsis [21]. Sepsis-induced AKI may cause multiple organ failure in critical ill patients and the mortality rate is high. Therefore, it is of important clinical significance to explore the prevention and control measures. Recent studies have shown that GA has a variety of protective effects on kidney. Based on the above, in this study, we evaluate the effect of GA on sepsis-induced AKI in rats.

We established the sepsis-induced AKI model by CLP in rats, which is one of the classical methods for sepsis and also the animal model most close to pathophysiological procedure of sepsis [22]. Kidney is the most sensitive organ in sepsis. After CLP, the serum Cre and BUN levels were significantly increased and renal tubule epitheliums were swelling with inflammatory cell infiltration in kidney, which finally resulted in AKI. Numerous studies have shown that mild loss of kidney function may increase the fatality rate of patients with sepsis [23]. So the early intervention in AKI with proper treatment will help reduce the mortality of sepsis patients. The preventive effect of GA against AKI was confirmed in our study by reducing pathological changes and improving renal function. Thus, we conclude that GA has real potential as a new medical treatment in sepsis-induced AKI. However, the possible mechanism of GA alleviating AKI was not clear, so we next performed various experiments to further explore it.

The essence of sepsis is the systemic inflammatory reaction and the inflammatory cytokines and mediators play important roles in pathogenesis of sepsis. TNF-*α*, IL-6, and IL-1*β* are the main inflammatory cytokines released in sepsis [24–26], which may assess the severity of sepsis. The cascade activation and interaction of inflammatory cytokines and mediators promote the formation of inflammatory cascade and finally lead to SIRS. In the present study, the productions of TNF-*α*, IL-1*β*, and IL-6 were significantly induced by AKI, which were suppressed by the treatment of GA. The inflammatory cytokine levels were consistent with the extent of renal injury, proving the anti-inflammatory effects of GA in vivo.

NO is derived from the oxidation of L-arginine, which is catalyzed by nitric oxide synthase (NOS). In sepsis, the expression of inducible nitric oxide synthase (iNOS) induced by inflammatory mediators and cytokines is significantly increased in immunocytes, such as neutrophils and macrophages. Researches have found that iNOS induced the production of NO and caused the oxidized stress in sepsis-induced AKI [27, 28]. Meanwhile, the studies about NOS inhibitor have confirmed the effect of iNOS on the development of AKI [29]. Study by Heemskerk et al. showed that selective iNOS inhibitor could prevent renal proximal tubule damage [30]. In the present study, the production of NO and expression of iNOS were significantly induced by AKI, which were suppressed by the treatment of GA. COX-2 is one of the downstream targets of NO and there is almost no expression in normal tissues. Under stimulation, COX-2 is largely expressed in immune cells and participates in and aggravates the inflammatory response. The level of COX-2 is associated with the severity of inflammation [31]. PGE_2_, an enzymatic product of COX-2, is an important inflammatory mediator and plays an important role in the inflammatory response [32]. Similarly, in our study, the production of PGE_2_ and expression of COX-2 were significantly induced by AKI, which were suppressed by the treatment of GA.

Recent studies have found that the apoptosis of renal tubular epithelial cells was the primary mechanisms of AKI. At early phase of sepsis, the number of apoptosis cells in kidney was significantly increased, which were closely connected with large releases of cytokines. Meanwhile, ischemic-reperfusion injury can promote the level of apoptosis in renal tubular epithelial cells. Study by Cunningham et al. showed that, in sepsis-induced AKI, apoptosis was found in kidney after 6 h of stimulation of LPS [33]. Jo et al. found that the apoptosis of renal tubular cells induced by inflammatory cytokines can be one of the possible mechanisms of renal dysfunction in endotoxemia [34]. Consistent with these results, obvious apoptosis induced by AKI was found in renal tissue in our study, which could be inhibited by GA treatment. Furthermore, we investigated the apoptosis signaling pathway through which GA could play a role in antiapoptosis. The apoptosis of renal tubular epithelial cells is induced through two major apoptosis signaling pathways, namely, the intrinsic mitochondrial pathway and the extrinsic death receptor pathway. Caspase-3, as a downstream component in the intrinsic and extrinsic apoptotic pathways, is an executioner caspase. The inhibition of caspase-3 activation can prevent cells from apoptosis. Bcl-2 family proteins are the most important apoptosis-related genes. Bcl-2 suppresses cell apoptosis via inhibiting the production of free radicals, decreasing mitochondrial membrane permeability, obstructing the release of cytochrome C, and activation of caspase. The ratio of Bcl-2/Bax determines the survival of the cells [35]. In our present study, GA significantly inhibited the activation of caspase-3 induced by AKI and increased the ratio of Bcl-2/Bax, which further confirmed the effect of GA against apoptosis.

NF-*κ*B is the key transcriptional regulatory factor of genes related to inflammation, which plays important roles in the development and progression of sepsis by inducing the transcription of inflammatory cytokines and starting the inflammatory cascade reactions [36]. In sepsis, the release of toxin and inflammatory mediators in the body can induce the degradation of I*κ*B and then free and activate NF-*κ*B. The activated NF-*κ*B quickly enters into nucleus across nuclear pore and induces the expression of TNF-*α*, IL-6, and so forth. The released cytokines can conversely activate NF-*κ*B and finally form cascade amplification effect of the positive feedback. So the activation of NF-*κ*B is key link of triggering excessive inflammatory responses. In experimental animal models of ALI, NF-*κ*B activation is increased [37]. Aosasa et al. found that the inhibitory effect of gabexate mesilate on the TNF-*α* production of activated human monocytes is mediated by the suppression of NF-*κ*B activation [38]. iNOS and COX-2 are two important proteins downstream from NF-*κ*B signaling pathway and the NF-*κ*B could specifically bind to the promoter sequence of iNOS and COX-2, which promotes the transcription of them and ultimately facilitates the production of NO and PGE_2_. Surh et al. found that COX-2 and iNOS were downregulated through suppression of NF-*κ*B activation [39]. A study by Moriyuki et al. demonstrated that the inhibition of NF-*κ*B signaling pathway restrained the catalytic function of COX-2 so that the production of PGE_2_ was decreased [40]. In the present study, the degradation of I*κ*B*α* and activation of NF-*κ*B were found in sepsis group, and the activation of NF-*κ*B was inhibited by GA treatment, suggesting that inhibition of NF-*κ*B suppressed the production of inflammatory cytokines and mediators induced by sepsis and played a key role in the protective effects of GA on AKI.

## 5. Conclusion

We have demonstrated the protective role of GA against sepsis-induced AKI by alleviating the pathological damage of kidney tissue, inhibiting the release of inflammatory cytokines and mediators, suppressing the kidney cells apoptosis, and inactivating NF-*κ*B. Our study indicates that treatment with GA might be a potential approach in the treatment of sepsis-induced AKI.
